# U-Shape Suppressive Effect of Phenol Red on the Epileptiform Burst Activity via Activation of Estrogen Receptors in Primary Hippocampal Culture

**DOI:** 10.1371/journal.pone.0060189

**Published:** 2013-04-01

**Authors:** Xu Liu, Ben Chen, Lulan Chen, Wan-Ting Ren, Juan Liu, Guoxiang Wang, Wei Fan, Xin Wang, Yun Wang

**Affiliations:** Neurology Department, Zhongshan Hospital, Institutes of Brain Science and State Key Laboratory of Medical Neurobiology, Fudan University, Shanghai, China; Universidade Federal do ABC, Brazil

## Abstract

Phenol red is widely used in cell culture as a pH indicator. Recently, it also has been reported to have estrogen-like bioactivity and be capable of promoting cell proliferation in different cell lines. However, the effect of phenol red on primary neuronal culture has never been investigated. By using patch clamp technique, we demonstrated that hippocampal pyramidal neurons cultured in neurobasal medium containing no phenol red had large depolarization-associated epileptiform bursting activities, which were rarely seen in neurons cultured in phenol red-containing medium. Further experiment data indicate that the suppressive effect of the phenol red on the abnormal epileptiform burst neuronal activities was U-shape dose related, with the most effective concentration at 28 µM. In addition, this concentration related inhibitory effect of phenol red on the epileptiform neuronal discharges was mimicked by 17-β-estradiol, an estrogen receptor agonist, and inhibited by ICI-182,780, an estrogen receptor antagonist. Our results suggest that estrogen receptor activation by phenol red in the culture medium prevents formation of abnormal, epileptiform burst activity. These studies highlight the importance of phenol red as estrogen receptor stimulator and cautions of careful use of phenol red in cell culture media.

## Introduction

Phenol red is a known pH indicator widely used in cell culture for detecting the pH change of the culture medium during the whole culture process. Currently, most of the commercially available culture mediums are sold with different phenol red concentrations, ranging from 15–45 µM [1]. However, whether phenol red has other than pH indicator function in the culture medium is still not fully understood. Phenol red has been reported to have a structural resemblance to certain nonsteroidal estrogens, and acts as a weak estrogen receptor stimulator [2]. In cell culture, it was reported to promote oestroblast proliferation [3], stimulation the human breast cancer-derived MCF-7 cells [1], [4], [5] and differentiation of bone marrow stromal cells [6], which were all due to its estrogen receptor stimulator property [1], [7].

In central nervous system, activation of estrogen receptors has been reported to affect the excitability of various types of neurons. 17-β-estradiol increases the excitability of gonadotrophin-releasing hormone neurons [8], medial vestibular nucleus neurons in brain stem [9] and hippocampal neurons [10] through either membrane or intracellular mechanisms. Estrogen has also been reported to decrease neuronal excitability by indirectly changing the local neurotransmitter release [11] particularly by changing the interaction with GABAergic neurons [12], [13]. In addition to the modulation of the neuronal excitability, activation of estrogen receptors could stimulate the spinogenesis [14], [15], [16] or affect the brain development by activating its two receptor subtypes: ERα and ERβ [17]. Since phenol red is a weak estrogen receptor stimulator [2] and also a pH indicator added in most of the culture medium, it is important to investigate whether phenol red might have direct modulatory effect on neuronal activity, which has never been explored so far.

In the current study, the effect of phenol red on the excitability of the cultured hippocampal neurons was investigated. Our results showed that without phenol red, abnormal epileptiform-like bursting activities were observed in most tested neurons in hippocampal cultures. Phenol red suppressed this epileptiform activity in an U-shape dose dependent manner, and the most effective dose was at 28 µM. This suppressive effect of phenol red was abolished by estrogen receptor antagonist ICI 182,780 [18], [19] and mimicked by the endogenous estrogen receptor agonist 17-β-estradiol. Our work suggests that activation of neuronal estrogen receptors is important to maintain normal neuron condition in primary culture.

## Methods

### Ethics Statement

All animal experiments were approved by the local committees of The Use of the Laboratory Animals, Fudan University and carried out in accordance with Chinese National Nature Science Foundation animal research regulation.

### Primary Hippocampal Neuronal Culture

Primary hippocampal neurons were prepared from embryonic day 18 Sprague Dawley rats similar as previously reported [20]. The pregnant rat was anaesthetized with chloral hydrate (400 mg/kg, i.p.), and pups were dissected out for tissue preparation. All the animals were then euthanized with over dose of chloral hydrate. After the dissection of the hippocampus, the tissue was rinsed in cold HBSS and then digested with 0.05% trypsin–EDTA for about 20 min at 37°C, followed by trituration with pipettes in the plating media (DMEM with 10% FBS, 10% F12 and 25 ug/mL penicillin/streptomycin). After rinsing twice, cells were counted and plated onto glass coverslips (22 × 22 mm; Carolina Biological Supply Co.) or a 35 mm petri-dish with 20 mm glass bottom well (Shengyou Biotechnology Co., Ltd) precoated with 0.1 mg/ml poly-D-lysine (Sigma-Aldrich, Co.). After culturing for 1 day, half of the media were changed into neuronal culture media (neurobasal media (GIBCO) containing 2 mM GlutaMAX™-I Supplement, 2% B27 and 25 u/mL penicillin/streptomycin) either without or with phenol red at 21.5 µM. Ara-C (2 µM; Sigma-Aldrich, Co.) was added 6–8 d after plating during the culture medium change, and cells were fed twice weekly thereafter. All cells were grown at 37°C and in 5% CO2. Unless stated otherwise, all tissue culture reagents were obtained from Invitrogen (San Diego, CA).

### Immunostaining Experiments

Immunohistochemistry experiment was performed according to previously reported from our lab [21]. Cells were fixed with 4% PFA (paraformaldehyde in 0.1 M phosphate buffer, pH 7.4) for 10–12 min. After several rinses in TBS (Tris-buffered saline, pH 7.4), cells were permeablized with 98% methanol for 1 min at −20°C and then blocked for 2 h in 10% NDS (normal donkey serum, Millipore) in TBS at room temperature. Subsequently cells were incubated at 4°C overnight with the following primary antibodies: rabbit anti-NeuN/Fox3 (NeuN, 1∶400; Biosensis, South Australia, Australia) and mouse-anti-GAD67 (1∶250; clone 1G10.2, Millipore, Billerica, MA). After several rinses in TBS, cells were incubated at room temperature with corresponding secondary antibodies: donkey anti-rabbit conjugated to Alexa Fluor 594 and donkey anti-mouse conjugated to Alexa Fluor 488 (1∶300; Molecular Probe). Cells were then rinsed several times in TBS for at least 30 min, mounted on slides and coverslipped with ProLong Gold antifade reagent (Molecular Probe).

### Electrophysiological Recordings

**Figure 1:**
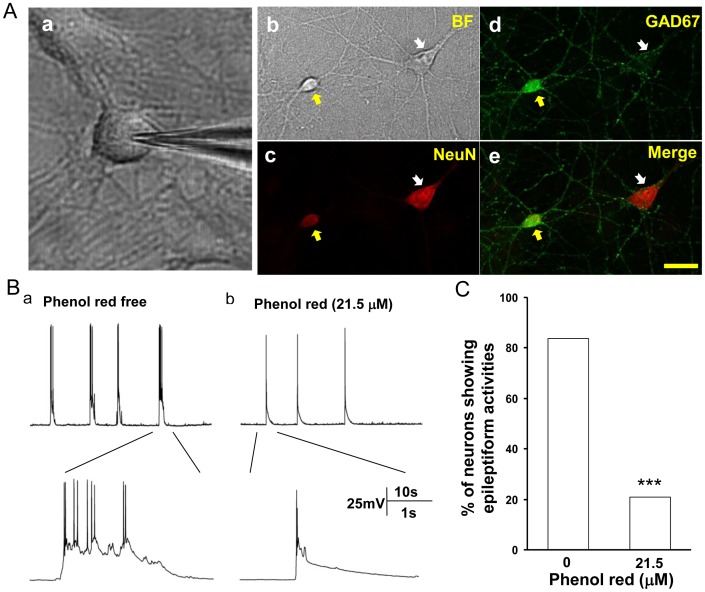
Phenol red suppressed epileptiform bursting in cultured hippocampal pyramidal neurons. A. (a) Bright field microscope picture showing an example of the pyramidal shaped neuron chosen for electrophysiological recordings; (b)–(e) pictures of immunohistochemical staining of cultured hippocampal neurons with (b, in bright field NeuN (c) and GAD67 (d). Pyramidal shaped neuron (white arrow) was GAD67 negative (d) and the oval shaped neuron (yellow arrow) was GAD67 positive (e). B. Current clamp recording traces from pyramidal neurons cultured in the media that was either phenol red free (a) or contained 21.5 micromoles of phenol red (b). In phenol red free medium, pyramidal cells generated repeated bursting of action potentials fired on top of the sustained membrane potential depolarization (a). In neurons cultured with phenol red, only single isolated action potentials riding on top of the EPSPs were recorded. C. Group analysis showed that the percentage of the bursting neurons in phenol red free culture medium (n = 19) was significantly higher than in phenol red added (21.5 µM) culture medium (n = 24, P<0.001). ***P<0.001.

The detailed *in vitro* electrophysiology protocol has been described previously [22], [23]. Briefly, pyramidal shaped culture neurons at DIV 14–17 were selected for recordings (Fig. 1Aa) as previously reported [22], [23]. Since our immunohistochemistry result has shown that the pyramidal shaped, but not those oval shaped, neurons were GAD67 negative (Fig. 1Ab–e), those pyramidal shaped neurons were chosen for the electrophysological recordings in current study. Whole-cell recordings were performed in current clamp mode using a MultiClamp 700B amplifier (Axon Instrument, USA). Patch pipettes (Sutter, USA) were pulled using Sutter P-97 (Sutter, USA) pipette puller and the final resistance of pipettes was 3–6 MΩ. The recording chamber was continuously perfused with a bath solution consisted of 128 mM NaCl, 30 mM Glucose, 25 mM HEPES, 5 mM KCl, 2 mM CaCl_2_, 1 mM MgCl_2_, pH 7.3 adjusted with NaOH, and the osmolarity was controlled between 305–315 mOsmol/l tested with Fisk Micro Osmometer (Model 210, Fisk Associates, USA). The pipette solution contained 125 mM KGluconate, 10 mM KCl, 2 mM EGTA, 10 mM HEPES, 10 mM Tris-phosphocreatine, 4 mM MgATP, 0.5 mM Na_2_GTP, pH 7.3 adjusted with KOH, and the osmolarity was controlled between 280–290 mOsmol/l. The series resistance was typically 10–20 MΩ and partially compensated by 30–50%. The membrane potential was held around −70 mV in current clamp mode. Data were acquired using pClamp 10 software (Axon Instrument, USA), sampled at 2–10 kHz, and filtered at 1 kHz. Off-line analysis was done with Clampfit 10 software (Axon Instrument, USA). A large depolarization shift was defined as a membrane potential up shift of ≥10 mV and lasting for ≥300 ms. A bursting activity was defined by at least five consecutive action potentials overlaying on the top of this large depolarization shift. When quantifying the percentage of neurons showing bursting activity, the criterion is at least two repeated bursts occurring during 10 min of recording [22], [23]. Data within a group were obtained from at least three different batches of cultured neurons.

### Drug Treatment Protocol

Hippocampal neurons were cultured with either commercial phenol red added (control group) or phenol red free neurobasal cell culture medium (testing group) throughout the whole culture period. In testing groups, different concentrations of the phenol red (10, 30, 60, 100 and 200 µM) or 17-β-estradiol (0.1 and 1 ng/ml) were added into the cell culture medium at the DIV 1. For antagonist study, ICI 182,780 (100 nM), an estrogen receptor antagonist [18], [19], was added to co-culture with phenol red at 30 µM.

### Data Analysis

Data were all presented as mean ± SEM and analyzed by SPSS13.0. Bell-shape curve fit was done with GraphPad Prism 4 (GraphPad Software, Inc.). Statistically significance between groups was determined using unpaired student *t*-test and chi-square test. Significance level was set at *P*<0.05.

## Results

### Effect of Phenol Red in Neurobasal Medium on Cultured Hippocampal Neurons

Phenol red has been widely used in cell culture as a supplement ingredient for pH change indicator. Phenol red was recently reported to promoter cell proliferation in cell cultures [4], but it has never been explored for its effect on neuron cultures. We first compared the neuronal discharges in hippocampal neurons cultured in a medium (neurobasal, NB27, GIBCO) with or without phenol red. Normal spontaneous discharges characterized as single or doublet action potentials fired on top of EPSPs randomly appeared in hippocampal neurons cultured in commercially available phenol red containing neurobasal medium (NB27, GIBCO, Phenol red at 21.5 µM) for 2 weeks (Fig. 1Bb), while bursting activities only occasionally appeared in minority of neurons (21%, 5 in 24). However, when neurons were cultured in phenol red-free neurobasal medium (NB27, GIBCO) for 2 weeks at DIV 14–16, the majority of neurons (84%, 16 in 19) had abnormal bursting activities characterized as at least 5 consecutive action potentials overlaying on top of a large depolarization shift (>10 mV) (Fig. 1Ba), which were similar with previous reported epileptiform bursting activities induced by convulsant stimulator [22], [23]. Statistic analysis indicated that neurons cultured in phenol red free medium had significantly more abnormal epileptiform spontaneous activities than those cultured in phenol red containing medium (P<0.001) (Fig. 1C).

### Dose-dependent Effect of Phenol Red on Epileptiform Bursting Activities in Cultured Hippocampal Neurons

**Figure 2:**
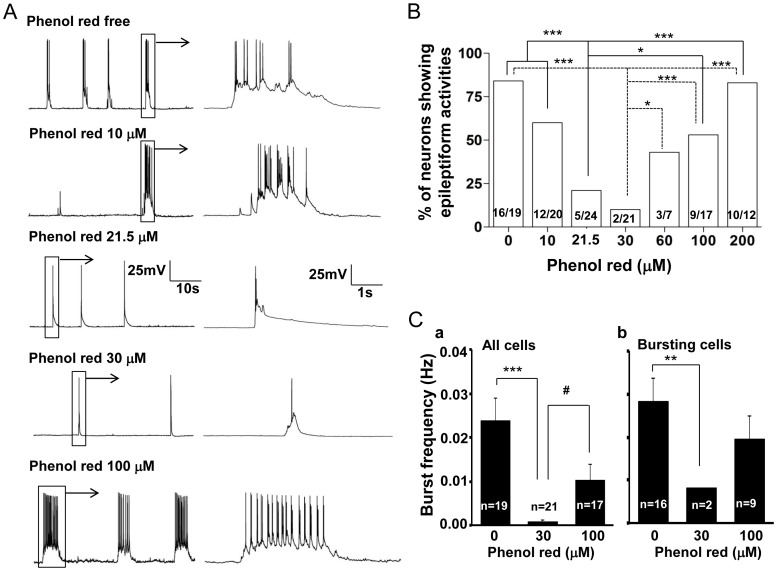
U-shape inhibition of the bursting activities in cultured hippocampal pyramidal neurons by phenol red. A. Current clamp traces showing hippocampal pyramidal neurons with different firing patterns in culture media with different phenol red concentrations (0, 10, 21.5, 30, 100 µM). Low and high concentrations of phenol red induced the most prominent and longer lasting depolarizations and bursting activity. B. Group data showing the U-shape suppressive effect of phenol red on the percentage of the bursting neurons in different concentrations of the phenol red (0–200 µM). C. Group data analysis showing the most significant decrease of the burst frequency by phenol red at 30 µM pooled either in all tested neurons (P<0.001) (a) or in only the bursting neurons (P<0.01) (b). **P<0.01, ***P<0.001; ^#^P<0.05.

We further studied whether phenol red had dose-dependent effect on the observed abnormal bursting activities in cultured hippocampal neurons. After 14 days treatment of phenol red at doses of 10, 30, 60, 100 and 200 µM, the percentage of neurons showing abnormal bursting discharges were suppressed by phenol red with U-shape response (Fig. 2A,B). Since the concentration of phenol red in commercially available NB27 culture medium is about 21.5 µM, we included this dose in our group data analysis. Our results demonstrated that, within the phenol red dose range we studied, the most effective dose to inhibit the abnormal bursting discharges was 30 µM (Fig. 2B). At this dose of phenol red, least neurons (9.5%, 2 in 21) exhibited bursting activities, which is significantly (P<0.001) lower than those cultured in phenol red free culture medium. At low phenol red doses (0, 10, 21.5 and 30 µM), there was a dose dependent inhibition of abnormal bursting activity with the proportion of the neurons had bursting activities at 84%, 60%, 21% and 9.5%, respectively (Fig. 2B). However, when the phenol red doses were higher than 30 µM the abnormal bursting discharges were again appeared in the majority of the recorded neurons. There was a dose dependent increase of the proportion of neurons (9.5%, 43%, 53% and 83%, respectively) that exhibited the abnormal bursting activities within the phenol red doses of 30, 60, 100 and 200 µM (Fig. 2B).

### Effect of ICI 182,780 on Suppressive Action of Phenol Red on Abnormal Activities in Cultured Hippocampal Neurons

**Figure 3:**
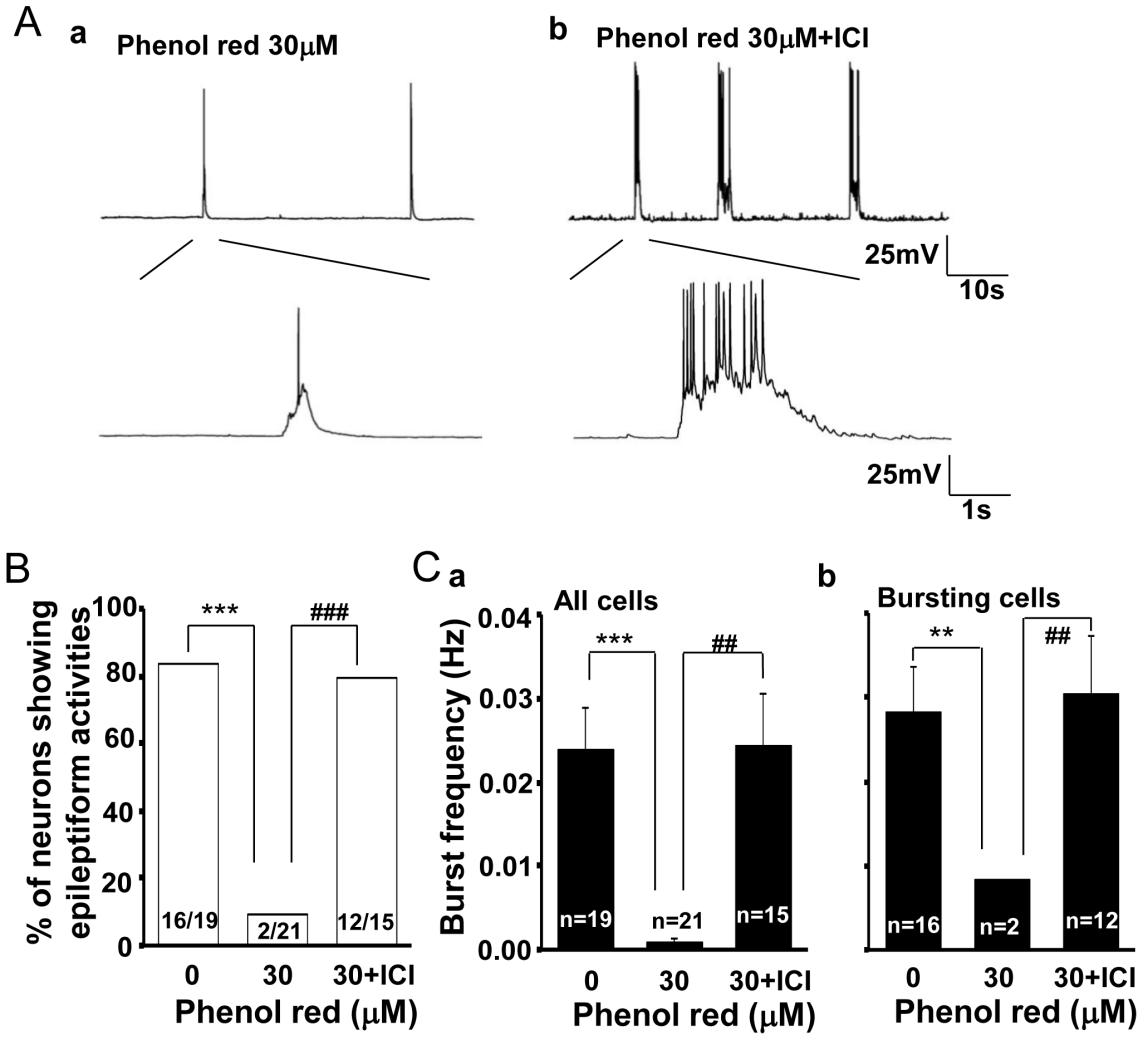
Estrogen receptor antagonist ICI 182,780 inhibits the suppressive effect of phenol red on the burst formation in cultured hippocampal pyramidal neurons. A. Electrical traces showing different spontaneous firing pattern in neurons cultured in medium containing either phenol red (30 µM) (a) or phenol red (30 µM) plus ICI 182,780 (100 nM) (b); B. Group data showing ICI 182,780 significantly (P<0.001) prevented the decrease of the percentage of the bursting neurons by phenol red at 30 µM; C. Group data analysis showing a significant blockade of the burst frequency by ICI 182,780 on the phenol red inhibition pooled either in all tested neurons (a) or in those bursted neurons (b). **, ^##^P<0.01; ***, ^###^P<0.001.

Since phenol red has been previously reported to have estrogen-like activity [1]–[7], we next used an estrogen receptor antagonist ICI 182,780 [17], [18] to test whether this suppressive effect of phenol red on abnormal bursting activities of the cultured neurons was mediated by the activation of the estrogen receptors. The result showed that ICI 182,780 (100 nM) significantly (P<0.001) inhibited the suppressive action of phenol red (30 µM) on the abnormal spontaneous epileptiform bursting activities (Fig. 3A). When cultured in 30 µM phenol red, only 9.5% (2 in 21) of the neurons showed epileptiform bursting activities; and, in contrast, 80% (12 in 15) of the neurons displayed the abnormal activities when neurons were co-cultured with phenol red (30 µM) and ICI 182,780 (100 nM) (Fig. 3B). There was no statistic difference of the percentage of the neurons showing bursting activities between the phenol red free group and 30 µM phenol red plus ICI 182,780 group (P = 0.254). This result suggests that the suppressive effect of phenol red on the abnormal epileptiform bursting activity of cultured neurons is likely due to the activation of the estrogen receptors.

### Effect of 17-β-estradiol on Hippocampal Neurons Cultured in Phenol Red Free Neurobasal Medium

**Figure 4:**
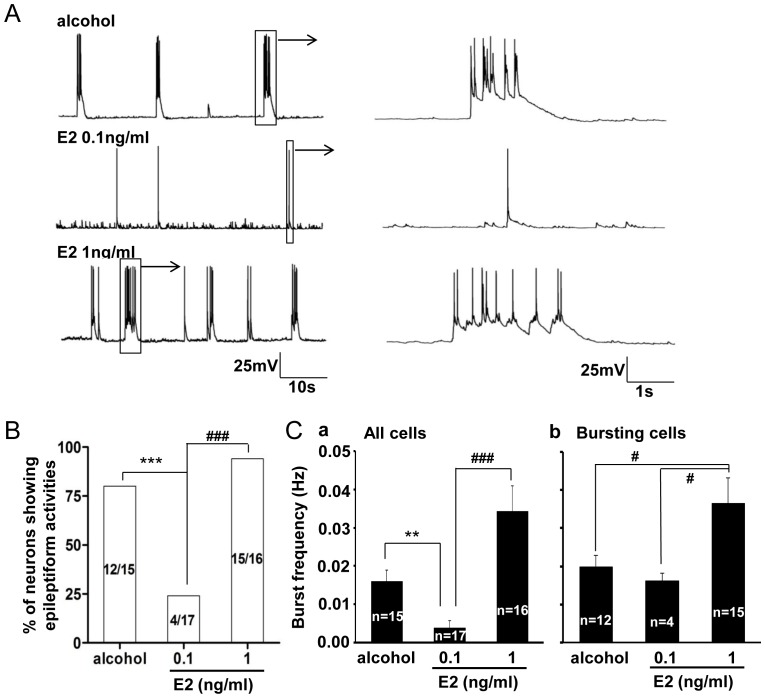
17-β-estradiol dose related inhibition of the epileptiform bursting activities in cultured hippocampal pyramidal neurons. A. Different firing patterns of neurons cultured in medium with either alcohol control or 0.1 and 1 ng/ml 17-β-estradiol. B. Group data showing the dose related suppressive effect of the 17-β-estradiol on the percentage of the neurons having the epileptiform bursting activities. C. Group data analysis showing a significant decrease of the burst frequency by 17-β-estradiol at 0.1 ng/ml pooled either in all tested neurons (P<0.01) (a) or a significant increase of the burst frequency in the estradiol at 1 ng/ml in the bursting neurons (P<0.05) (b). **P<0.01, ***P<0.001; ^#^P<0.05, ^###^P<0.001.

We further studied whether direct stimulation of estrogen receptors with endogenous estrogen receptor agonist 17-β-estradiol had similar effects as those seen in phenol red experiments. In phenol red free neurobasal culture medium containing alcohol (vehicle control for 17-β-estradiol), 80% (12 in 15) of recorded neurons exhibited bursting activities, which was similar to that in non-alcohol added control medium (84%, 16 in 19). However, the percentage of neurons showing epileptiform activity was reduced to only 24% (4 in 17) in 17-β-estradiol (0.1 ng/ml) added cultural medium (P<0.001) (Fig. 4 A, B). Similar as that of phenol red, the percentage of the neurons showing abnormal epileptiform bursting activities was again increased (94%, 15 in 16) when the 17-β-estradiol concentration was raised to a high dose at 1 ng/ml, which was significantly (P<0.001) more than that of 17-β-estradiol at 0.1 ng/ml dose group (Fig. 4A, B). However, the high concentration of 17-β-estradiol (1 ng/ml) treated group had no significant difference in comparison with those in the alcohol control group (P = 0.254). This result indicates that, similar with phenol red, the estrogen receptor agonist 17-estradiol also exhibited a dose-dependent effect on the spontaneous epileptiform bursting activity. Low concentration (0.1 ng/ml) blocked epileptiform bursts, but higher concentration (1 ng/ml) had no effect.

### Effect of Phenol Red and 17-β-estradiol on the Abnormal Epileptiform Bursting Properties

We further analyzed the effect of both phenol red and 17-β-estradiol on the bursting frequency of those recorded neurons. The results demonstrated that both phenol red and 17-β-estradiol also had dose related effect on bursting frequency in those bursting neurons. The burst frequency (Hz) in different tested phenol red concentration groups (0, 30, 100 µM) was 0.028±0.005 (n  = 16), 0.008 (n  = 2, P = 0.002 in comparison with the control group), 0.020±0.005 (n  = 9, P = 0.257 in comparison with the control group P = 0.072, in comparison with the phenol red 30 µM group), respectively (Fig. 2C). Estrogen receptor antagonist ICI 182,780 (100 nM) reversed this suppressive effect of phenol red (30 µM) on the abnormal epileptiform bursting activities in cultured hippocampal neurons (P = 0.008; Fig. 3C). Similarly, the burst frequency (Hz) in either alcohol vehicle control or 0.1 or 1 ng/ml 17-β-estradiol treated groups was at 0.020±0.003 Hz (n = 12), 0.016±0.005 Hz (n = 4, P = 0.533 in comparison with the alcohol control group) and 0.037±0.007 Hz (n = 15, P = 0.034 in comparison with the alcohol control group, and P = 0.028 in comparison with the 0.1 ng/kg group), respectively (Fig. 4C). This result suggests that phenol red, by activation of estrogen receptors, not only affects the proportion of the neurons to have the bursting activities, but also dose-dependently suppresses the abnormal epileptiform bursting frequency.

### Comparison between Commercial Available Phenol Red Concentration (21.5 µM) and 30 µM Phenol Red on the Bursting Properties in those Bursting Neurons

**Figure 5:**
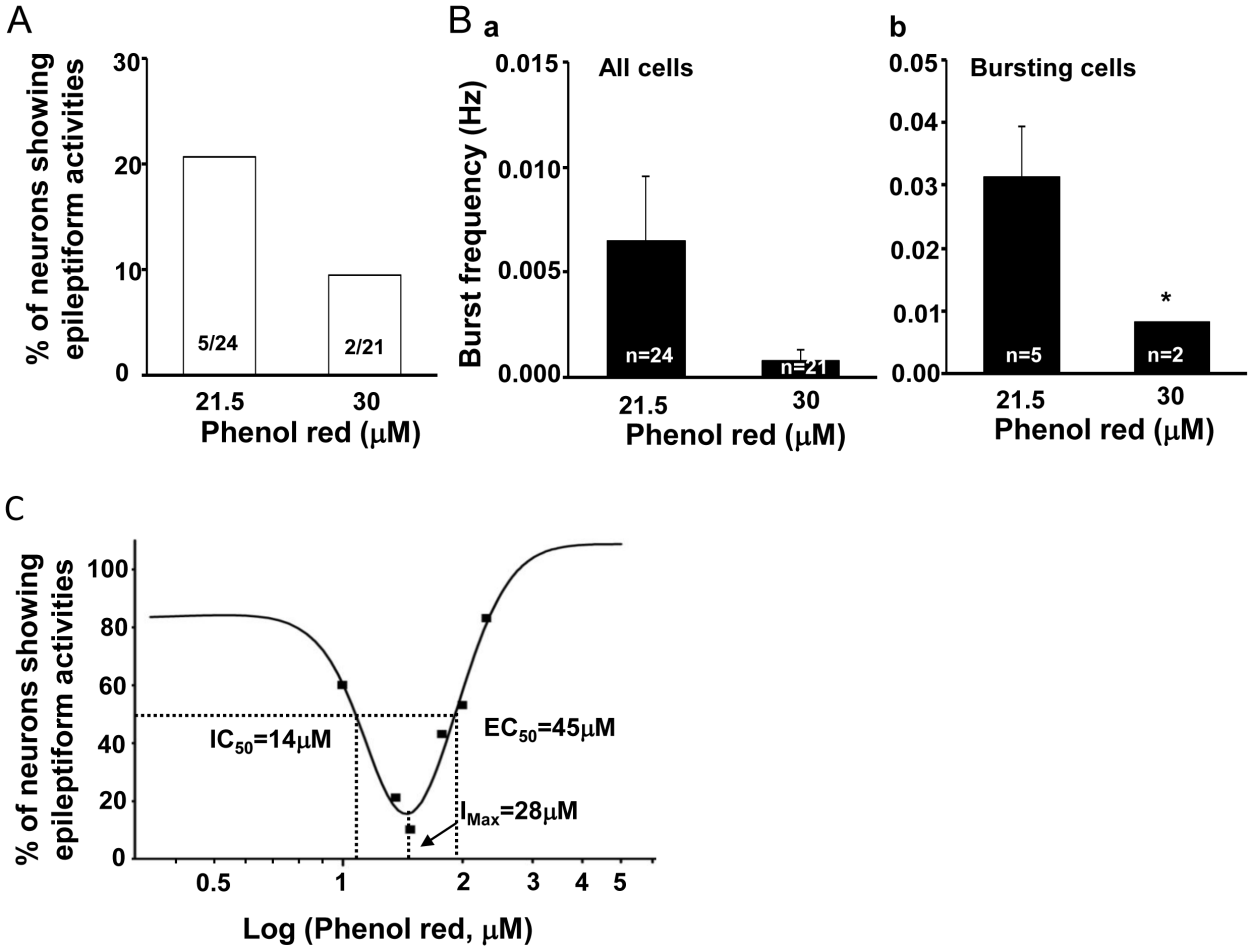
Effect of phenol red concentration at 21.5 µM and 30 µM in the burst activities in the cultured hippocampal pyramidal neurons. A–B. Bar histograms showing stronger suppressive effect of phenol red at 30 µM on the epileptiform bursting activities than at the commercially available medium with the added concentration of phenol red (21.5 µM). C. Curve of the two phase effect of phenol red on the epileptiform bursting activities with the inhibitory phase at the low concentration (IC_50_ = 14 µM) and the enhancing phase at the high concentration (EC_50_ = 45 µM); with the most effective concentration for the burst suppression seen at 28 µM. * P<0.05.

In current study, the phenol red added culture medium supplied by the GIBCO was with phenol red concentration at around 21.5 µM (GIBCO technique reference). However, our dose dependent experiments suggested that phenol red at 30 µM had better suppressive effect than phenol red at 21.5 µM in commercial available culture medium. The proportions of neurons that had bursting activities were 21% and 9.5% in 21.5 µM and 30 µM phenol red, respectively (Fig. 5A). In addition, the frequency of bursting activity in those two groups was 0.031±0.008 Hz (n = 5) and 0.008±0.000 Hz (n = 2), respectively (P = 0.044) (Fig. 5B). This result suggests that the current commercial available neurobasal culture medium might not contain the most effective concentration of phenol red, at least for neuronal culture, to maintain the best physiological condition of the cultured neurons.

We further calculated, by using Bell-Shape Response Curve Fit (GraphPad Prism 4, GraphPad Software, Inc.), the dose response of the phenol red suppressive effect on abnormal bursting activities in cultured hippocampal neurons. The calculated IC_50_ (between 0–30 µM) and EC_50_ (between 30–200 µM) was 14 µM and 45 µM, respectively; and the best phenol red concentration calculated for maintaining the normal cultured hippocampal neuron activity was 28 µM (Fig. 5C), which is different to the phenol red concentration (21.5 µM) in current commercial supplied phenol red added Neurobasal medium used in current study. This result suggests that the phenol red concentration at 28 µM, rather than 21.5 µM in current commercial available culture medium, may be more suitable for maintaining the physiological condition in neuronal cultures.

## Discussion

In the current study, we demonstrated that phenol red, in addition to its wildly used property as a culture medium pH indicator, also had suppressive effect on abnormal epileptiform bursting activities in cultured hippocampal pyramidal neurons. This effect is likely due to the activation of estrogen receptors, since 17-β-estradiol, an estrogen receptor agonist, could mimic this phenomenon, and ICI 182,780, an estrogen receptor antagonist, inhibited the suppressive effect of phenol red on the abnormal epileptiform burst activities in cultured hippocampal neurons.

### The Abnormal Epileptiform Bursting Activities in Phenol Red Free Hippocampal Cultures

In this current study, we first demonstrated that there was a significant more proportion of the cultured hippocampal neurons having abnormal epileptiform bursting activities when cultured in phenol red free medium than those in phenol red containing medium. The abnormal epileptiform bursting activities have been reported to exist not only in primary neuron culture [22], [23], but also in organotypic slice culture [24], [25]. However, this epileptiform activities were normally occuring only in the minority of the neurons [22], [23], and likely were related to the age in the culture system [24], [25]. The propotion of the neurons to have the spontaneous epileptiform bursting activities in our current phenol red containing culture condition (neurobasal culture medium from GIBCO with added phenol red at 21.5 µM) was only at around ∼20%, which was similar to that of previously reported [22], [23] in phenol red free but fetal bovine serum added culture medium. However, our results showed that this abnormal epileptiform bursting activity occurring rate was significantly lower than that in phenol red free NB27 culture medium (>80%, see Fig. 1). Cell culture system is divided into, in terms of the culture medium usage, fetal bovine serum added and non-added system. Fetal bovine serum is a nature product which contains variety of cultured cell needed molecules/proteins. Non- fetal bovine serum added culture medium has also been widely used for cell culturing, including neuronal culture, such as MEM, NMEM, neurobasal, etc. However, they would be possibly lacking of certain important molecules/proteins naturally contained in the fetal bovine serum, in which estradiol might be one of them. Since most of the cell culture media are phenol red added ones for the purpose of pH indication, the cultured cells are likely to be constantly stimulated through the activation of the estrogen receptors due to the estrogen receptor stimulator property of the phenol red [1]–[7]. Thus our result, the significant difference between the neurons to have bursting activities in either phenol red-free or phenol red-containing culture medium, suggests that estrogen receptors may play an important role in modulating the firing properties of the cultured neurons.

### Phenol Red U-shape Dose Related Inhibits the Epileptiform Bursting Activities of the Cultured Hippocampal Pyramidal Neurons *in vitro*

Phenol red is widely used in cell culture medium as a pH indicator, because of its color sensitivity at pH range of 6.8 to 8.2. Within this pH range, its color exhibits a gradual transition from yellow to red. In culture medium, phenol red shows in pink at normal physiological pH (pH 7.3–7.4), and it turns to yellow while the pH is lower than 7.0 and to bright pink while the pH is above 8.2. Thus, the change of the pH in cell culture medium was easily detected by directly observing the color change. Although previous studies have showed that phenol red could promote cell differentiation in culture [3], [4], [6], [7], the current study was the first to provide the experimental evidence that phenol red also has the physiological function in modulating neuronal firing during culture process. By using patch clamp techniques, we recorded the spontaneous neuronal action potential in hippocampal pyramidal neurons and used epileptiform burst firing as the functional biomarker to detect whether phenol red has functional effect on these neurons. Our dose dependent experiments showed that phenol red had U-shape like suppressive effect on the abnormal epileptiform bursting activities:- phenol red at the doses range between 0–30 µM had dose dependent inhibitory action with the IC_50_ at 14 µM; and when the phenol red dose range increased to over the 30 µM, the abnormal epileptiform bursting activities were reappeared with dose dependent manner with the EC_50_ at 45 µM. This result indicated that phenol red had dual modulation effect on abnormal neuronal bursting activities, likely with suppressive effect at the low concentration range and the enhancing effect at the high concentration range. Although our further estrogen receptor agonist and antagonist experimental data suggest that this suppressive effect of phenol red at the low-concentration range on the epileptiform bursting activities is due to the weak estrogen receptor stimulator property of the phenol red [2], the reason why phenol red has this U-shape property on the neuronal bursting activities in culture is still unknown. We hypothesis that it may be, at least in part, due to the subtype specific of the estrogen receptors in the hippocampus.

### Involvement of Estrogen Receptors in Phenol Red Suppressing Epileptiform Bursting Activities in Hippocampal Culture

Recently, phenol red, in addition to its pH indicator property, has also been found to have structural resemblance to certain nonsteroidal estrogens, and acts as a weak estrogen receptor stimulator with the binding affinity to the estrogen receptors at around 0.001% of the estradiol [1], and can mimic the bio-effect of estradiol through stimulating of the estrogen receptors [1], [2], [4], [26], [27]. Estrogen is a main hormonal of the female mammals (but also exists in males), which has a complex and wide physiological and pathophysiological effect, such as promoting cell proliferation [14], [15], [16] as well as modulating the neuronal excitability [9], [10], [28], [29], [30], [31]. As an estrogen receptor stimulator, phenol red, besides to have cell differentiation promoting properties in cell culture [1], [2], [3], [4], [6], may also have the modulating effect on the neuronal excitability by activation of the estrogen receptors. Indeed, our results showed that 17-β-estradiol, an estrogen receptor agonist, mimicked the dose related suppressive effect of the phenol red on the epileptiform bursting activities (Fig. 4), and the estrogen receptor antagonist ICI 182,780 [18], [19] blocked the suppressive effect of the phenol red (Fig. 3). These results confirm that the phenol red to suppress the bursting activities in cultured hippocampal neurons is due to the activation of the estrogen receptors. However, from our current study, we could not distinguish which subtype of the estrogen receptors has mediated this function, since ICI 182,780 is neither α nor β estrogen receptor subtype selective antagonist. The involvement of either α or β subtype estrogen receptors in mediating this phenol red action needs to be further studied by using α and β estrogen receptor subtype selective antagonists in future. Since estrogen receptor stimulation could either enhance the neuronal excitability by directly modulating the intrinsic properties [10], [29] or, in the other direction, decrease the neuronal excitability by indirectly changing the local neurotransmitter release [11], [30] particularly by interaction with GABAergic neurons [11], [12], our current experimental results could not reveal whether the inhibitory action on the neuronal bursting activities of the phenol red by activation of the estrogen receptor is direct or indirectly on the recorded pyramidal neurons. Further investigation on this aspect is also required.

### Functional Implication of the Phenol Red Usage in the Neuroal Culture

The results from our current study suggest that estrogen receptor activation is important to maintain the normal neuronal physiological conditions, at least on the firing properties of the neurons, during neuronal cell culture. Neuron is an excitable cell, in which the action potential is an important feature property of its excitability and transmitting of the excitation. Abnormal action potential firing such as epileptiform burst firing, caused by varies reasons such as convulsant stimulation [17], [22], would induce abnormal epileptiform activity dependent cellular cascade change even to trigger cell apoptosis [22], [32]. We indeed observed in our current study that, without estrogen receptor stimulation during the culture process in phenol red free medium, cultured hippocampal neurons were having high probability to have epileptiform burst firing. Thus, our result suggests that neurons cultured in phenol red or estradiol free culture medium for long time might be likely in a non-physiological condition.

Our dose response experiment results further demonstrated that phenol red exhibited U-shape like suppressive effect on the abnormal bursting activity of the cultured hippocampal neurons, and the most effective calculated phenol red concentration, after the bell-shape response curve fit, is 28 µM (Fig. 5). Either lower or higher than this concentration (28 µM), more neurons tended to have abnormal bursting activities and also with higher bursting frequency. To our surprise, we have discovered that in the current commercial available phenol red added culture medium, the phenol red concentration are in a random order, varies in a range of 15–45 µM [1], [4]. The phenol red concentration is 15 µM for RPMI 1640 medium, 21.5 µM for Neurobasal, 27 µM for MEM, 30 µM for Eagle's MEM and improved MEM, and 45 µM for DMEM. Even from a same commercial provider, such as GIBCO, the culture medium contains different concentration of the phenol red, for example at 21.5 and 27 µM for either Neurobasal or MEM, respectively. The reason, as we hypothesis, is that phenol red has only been considered as a pH indicator in those commercial available culture medium. Although we have not tested the spontaneous neuronal activities on the neurons cultured in all those culture medium systems, results from our current study still high likely to indicate that neurons were probably not in a healthiest physiological condition when cultured in those commercial available culture mediums. Thus, we suggest that, in future, phenol red concentration in those bovine serum free cell culture medium should have a concentration at a constant 28 µM, in which it may most effectively maintain the normal spontaneous firing condition for those cultured neurons. In addition, when the phenol red has to be added into bovine serum added culture medium as a pH indicator, the influence of the phenol red as an estrogen receptor stimulator also can not be ignored, and its amount should be carefully calculated to add on top of the serum containing estrogen effect.

### Conclusion

In conclusion, the results from this current study demonstrated that estrogen receptor stimulation is important to maintain the physiological firing condition in neuronal culture. Different phenol red concentrations in fetal bovine serum free culture medium had, in addition to its pH indicator property, U-shape like suppressive action on abnormal epileptiform bursting activities by its estrogen receptor stimulator property. Thus, the influence of the phenol red in the culture medium can not be ignored and need to be used cautiously in future. And our results suggest that the most effective phenol red concentration to inhibit the abnormal epileptiform bursting activities is 28 µM. This concentration should be used as a guide concentration for future cell culture system, at least for neuronal culture.
